# Historical Vignette on Trepanation: The Evolution of Opening the Skull

**DOI:** 10.7759/cureus.117427

**Published:** 2026-10-04

**Authors:** Dilmareth E Natera-Rodriguez, Elizabeth Harty, Andrew Grande

**Affiliations:** 1 Neurosurgery, University of Minnesota, Minneapolis, USA; 2 Medicine, University of Minnesota, Minneapolis, USA

**Keywords:** ancient surgery, craniotomy, historical vignette, history of neurosurgery, medical history, neurosurgery history, neurosurgical techniques, skull surgery, surgical innovation, trepanation

## Abstract

Trepanation, the ancient practice of drilling or scraping a hole into the human skull, is one of the earliest known surgical procedures. Its evolution, from prehistoric times to modern neurosurgery, reflects the development of medical knowledge and surgical techniques, depicting significant progress and cultural variations that have persisted until our modern era. From its origins in Neolithic societies, where it was used for therapeutic and ritualistic purposes, to its refinement in ancient Greece, medieval Europe, and beyond, trepanation has been crucial to the history of medicine. Its legacy is seen in modern neurosurgical techniques, reflecting humanity’s enduring quest to understand and treat cranial conditions.

## Introduction and background

Long before anesthesia, antisepsis, neuroimaging, or written anatomical knowledge, human communities discovered that the skull could be deliberately opened and that some individuals survived the procedure [1-3]. Healed cranial defects from multiple archaeological settings show that trepanation was not a single isolated experiment, but a recurring surgical practice developed in different regions and periods. The historical importance of trepanation therefore lies not merely in the creation of a hole in bone; it lies in the enduring challenge of gaining access to the intracranial compartment while limiting injury to the brain, meninges, vessels, and surrounding tissues [1-4].

Trepanation, also termed trephination or trephining, generally refers to the intentional perforation or removal of a circumscribed portion of the cranial vault. Modern terminology is more specific: a craniotomy involves temporary removal of a planned bone flap that is ordinarily replaced after the intracranial procedure, whereas a craniectomy leaves the bone flap unreplaced, commonly to accommodate swelling or facilitate decompression [5]. These distinctions reflect a level of operative planning that emerged only after centuries of progress in anatomy, anesthesia, antisepsis, hemostasis, and localization.

The meaning of trepanation has never been singular. Depending on the population and period, scholars have interpreted it as a treatment for cranial trauma, a response to neurological or behavioral symptoms, a ritual or symbolic act, or some combination of these possibilities [1,3]. Skeletal evidence can show the morphology of an opening and, when remodeling is present, that the patient survived long enough for healing to occur. It cannot by itself identify the operator’s intention, the patient’s preoperative condition, or the functional outcome. Historical interpretation must therefore distinguish direct material evidence from plausible but unprovable explanations.

The objective of this narrative historical vignette is to examine how opening the skull evolved from prehistoric trepanation to modern craniotomy, with particular attention to progressive control of surgical indication, anatomical risk, pain, infection, hemorrhage, lesion localization, and perioperative physiology. The six periods used below are organizational categories rather than uniform or strictly linear stages. Knowledge was developed, transmitted, modified, and at times lost or rediscovered across different cultures. The central argument is that the decisive transformation in cranial surgery was not simply learning that the skull could be opened; prehistoric practitioners had already demonstrated that possibility. The deeper transformation was the gradual control of the hazards surrounding that act [4].

A part of this work was select as an E-poster for the American Association of Neurological Surgeons (AANS) annual meeting, held in Boston from April 25 to 28, 2025.

## Review

Methods

This article was designed as a narrative historical vignette rather than a systematic review or meta-analysis. No protocol was registered. The domains informed the reporting and revision strategy emphasized in the Scale for the Assessment of Narrative Review Articles (SANRA), including explicit statement of the review’s importance and aims, description of the literature search, appropriate referencing, scientific reasoning, and contextual presentation of evidence [6]. SANRA was used as a quality framework rather than as a claim of systematic-review methodology.

Candidate sources were identified through targeted searches of PubMed/MEDLINE, Google Scholar, Crossref, and reference lists of relevant historical and clinical publications using combinations of the terms trepanation, trephination, craniotomy, craniectomy, cranial surgery, skull surgery, and history of neurosurgery, together with period-, technique-, culture-, and figure-specific terms such as Hippocrates, Galen, Al-Zahrawi, Avicenna, Vesalius, Ambroise Paré, Harvey Cushing, and Walter Dandy. No date restriction was applied because of the historical scope. Sources in English and selected French, German, and Hungarian literature were considered when accessible. The literature was updated through September 2026, including recently published historical reviews and paleopathological studies [4,7].

Because the topic spans archaeology, paleopathology, classical and medieval texts, biography, instrument history, and modern clinical neurosurgery, a single reproducible database strategy would not adequately capture all relevant source types. Sources were therefore selected for their relevance to major historical transitions and, when possible, preference was given to peer-reviewed publications, primary historical texts, and directly verifiable scholarship. No formal risk-of-bias instrument or quantitative synthesis was performed.

Quantitative archaeological findings are reported descriptively rather than pooled. Sample size, historical period, and the method used to infer survival are provided whenever the source permits. Importantly, “survival” in paleopathological studies generally refers to evidence of osseous healing after trepanation and is not equivalent to prospectively measured perioperative survival, neurological recovery, or functional outcome.

Neolithic era: prehistoric origins of trepanation

Trepanation is documented in prehistoric populations and is widely regarded as one of the oldest known surgical procedures [1-3]. Archaeological specimens show several methods of cranial opening, including scraping, grooving, drilling, and combinations of multiple perforations connected to remove a segment of bone. The morphology of the defects indicated that the technique varied by place, period, available tools, and local practice. Recent analysis of 41 Neolithic trepanned skulls from French museum collections further demonstrated substantial variation in the size and geometry of cranial openings, reinforcing that prehistoric trepanation was technically heterogeneous rather than a single standardized maneuver [7].

Interpretation of the indication remained more difficult than the identification of technique. Trauma was a plausible indication in many specimens, particularly when cranial injury was associated with the opening, but ritual, neurological, and symbolic explanations have also been proposed [1,3]. The important methodological point is that these explanations cannot be assigned solely from the presence of a healed defect. Healing establishes biological survival after bone injury; it does not prove why the procedure was undertaken.

Archaeological series nevertheless demonstrated that survival after trepanation could be substantial. In Late Iron Age Switzerland, Moghaddam and colleagues reviewed published trepanations and reported a 78% survival rate based on evidence of healing in that regional material [2]. The largest quantitative historical comparison came from Peru. Kushner and colleagues analyzed data from more than 800 trepanned crania spanning nearly two millennia. Long-term survival inferred from osseous healing was approximately 40% in the earliest period (400-200 BCE), and it increased to as high as 91% in samples from approximately 1000-1400 CE, and averaged 75%-83% during the Inca period [3]. These values are striking, but they remain osteological estimates rather than modern clinical outcome rates.

The Peruvian evidence is particularly instructive because it suggests technical learning over time. Openings became smaller and the practice appeared to have favored approaches that minimized dural violation, a feature Kushner and colleagues proposed as one contributor to improved survival [3]. The broader lesson is not that prehistoric surgery was “safer” than modern surgery, but that empirical technique could be refined through repeated experience even in the absence of written neuroanatomy, anesthesia, antibiotics, or physiological monitoring.

Prehistoric trepanation therefore established the first durable principle in the history of cranial access: the skull was surgically penetrable, and survival depended in part on how the opening was made. Later eras would add increasingly explicit knowledge of why, where, when, and under what physiological conditions such access should occur (Figure 1).

**Figure 1:**
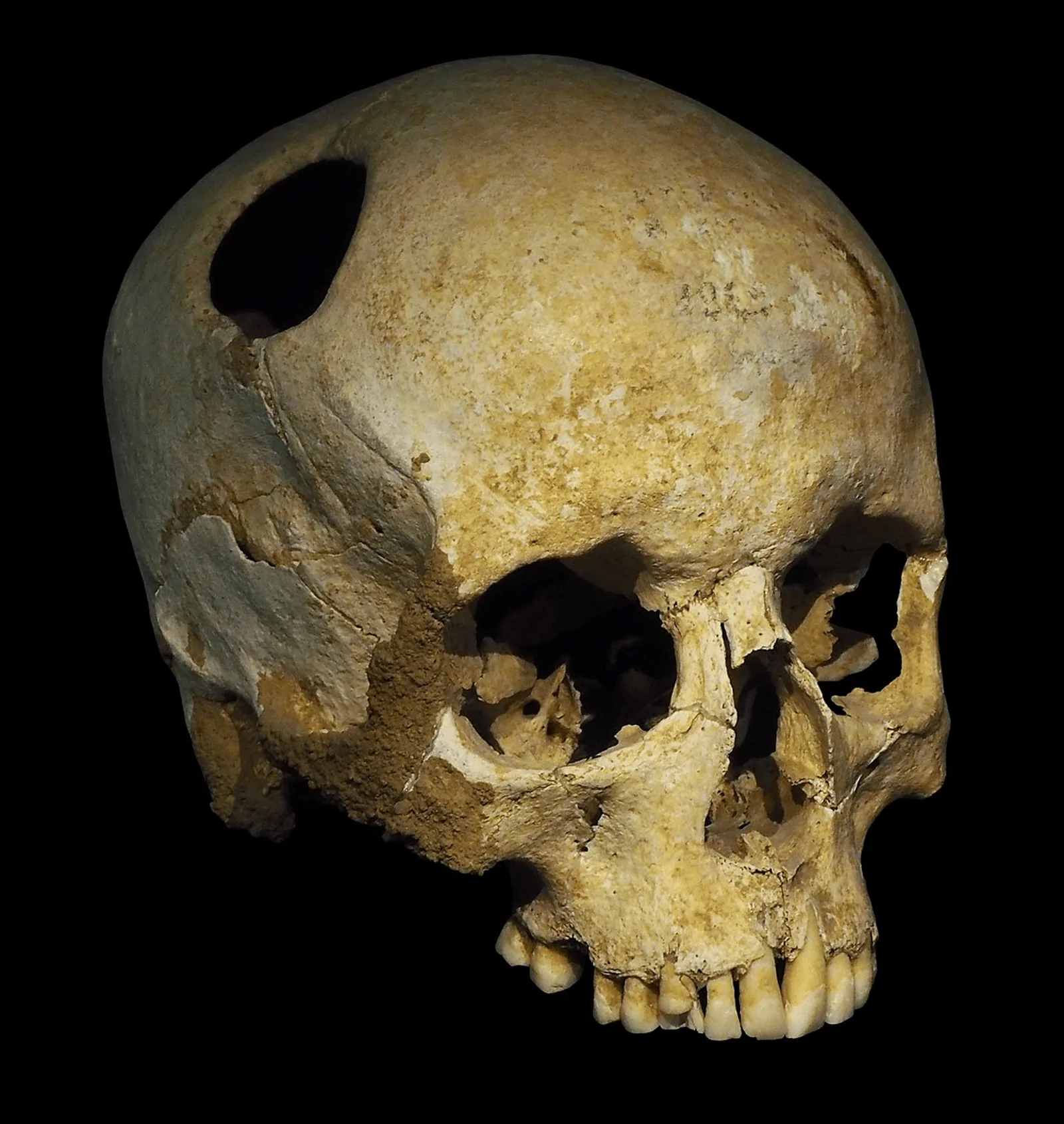
Trepanned skull of a woman from Corseaux (ca. 3500 BCE), with healed margins indicating survival. Image credit: Photograph by Rama, sourced from Wikimedia Commons, used under Creative Commons Attribution-ShareAlike 3.0 France license.

Classical antiquity: from material practice to written surgical reasoning

In ancient Egypt, the Edwin Smith Papyrus - copied in the second millennium BCE from earlier material - offered one of the earliest surviving examples of structured clinical reasoning for cranial and spinal trauma. Its 48 cases were organized by examination, diagnosis, prognosis, and management. The text described the brain, meninges, cerebrospinal fluid, cranial fractures, and neurological findings with a degree of observational discipline that is remarkable for its period [8]. Although the papyrus was not a manual of elective trepanation, it demonstrated an important shift toward clinical classification of head injury.

Greek medicine provides the clearest surviving written account of trepanation as a defined surgical intervention. In 'On Injuries of the Head,' Hippocratic authors described cranial fracture patterns, clinical examination, and techniques for trepanation, while emphasizing caution around sutures and depth of drilling [9,10]. The significance of this literature was not that every recommendation corresponds to modern practice; some indications reflected contemporary pathophysiological assumptions. Its importance lay in the transformation of trepanation into a procedure discussed in relation to injury pattern, anatomy, technique, and anticipated risk.

Archaeological evidence from Greece provided an important counterpoint to the texts. The trepanned skull from Chios bore a parietal opening associated with a linear fracture and evidence of survival, and has been interpreted in relation to Hippocratic teaching [9]. A larger analysis of trepanations from ancient Greece, including new cases from Akanthos, documented drilling and scraping techniques and found evidence supporting cranial trauma as a major indication in many cases. That study reported a survival rate of 62.9% across its compiled Greek material, while also emphasizing limitations of skeletal representativeness and preservation [11].

Galen and later Greco-Roman authors continued to discuss cranial anatomy, trauma, and specialized instruments [10]. The instrument tradition itself became increasingly sophisticated. Circular trepans or trephines, guide pins, elevators, and other devices allowed surgeons to control the depth and geometry of bone removal more deliberately. Recent historical analyses of trepanation technique and cranial sutures underscore how concerns about plunging, dural injury, and suture anatomy persisted for centuries and shaped the evolution of the instrument [12,13].

Classical antiquity therefore contributed something distinct from prehistoric technical success: explicit surgical reasoning. Trepanation became increasingly linked to categorized injuries, described anatomy, named instruments, and written recommendations. That intellectual structure would later be transmitted, translated, contested, and expanded across the medieval Mediterranean world.

Medieval period: preservation, transmission, and refinement

After antiquity, cranial surgery continued across Europe and the Islamic world within different scholarly and operative traditions. Physicians of the Islamic Golden Age preserved and critically developed elements of Greek and Roman medicine. Avicenna’s Canon of Medicine discussed head injury and cranial fracture management, contributing to the transmission of surgical knowledge through Arabic scholarship and later Latin translation [14].

Al-Zahrawi (Albucasis, 936-1013 CE) is especially important because his Kitab al-Tasrif described operative instruments and techniques. A recent re-examination of the surgical chapters, including translation from Arabic, highlighted his management of cranial and spinal trauma and his descriptions of trephination and related instruments [15]. This evidence is more directly relevant to Al-Zahrawi’s neurosurgical practice than secondary summaries that merely mention medieval instrumentation.

In medieval Europe, trepanation remained closely associated with cranial trauma, depressed fractures, and drainage. Surgical authors inherited classical concerns regarding depth, the dura, and wound contamination, while instrument design continued to evolve. Historical analysis of the later Middle Ages described increasing standardization of narrow, non-plunging trepans and enlargement of cranial openings through multiple holes connected by chisels [12].

Archaeological series again showed that some patients survived. In southwestern Germany, seven of eight trepanned individuals from sixth- to eighth-century burials demonstrated extensive healing, although the small denominator limits generalization [16]. A Hungarian review compiled 115 trephined skulls across multiple archaeological periods. The authors reported long-term survival of 63% among pre-Hungarian cases and 84% among tenth-century Hungarian cases, while also noting that the majority of their material derived from 10th-century contexts [17]. These values should not be treated as directly comparable clinical survival rates because case ascertainment, chronology, preservation, and criteria for healing differ across archaeological populations.

The medieval period was therefore best understood as a bridge rather than a static interval. Knowledge of cranial opening persisted, instruments became increasingly specialized, and Arabic and Latin scholarship helped transmit earlier concepts into the surgical cultures that would flourish during the Renaissance [18].

Renaissance: anatomy, instruments, and direct observation

The Renaissance transformed surgery by changing the authority on which anatomical knowledge rested. Andreas Vesalius’s De Humani Corporis Fabrica (1543) exemplified the shift toward direct anatomical observation and dissection. For cranial surgery, improved understanding of the calvaria, sutures, meninges, and intracranial relationships provided a more reliable spatial framework for operative planning than inherited text alone [19].

Ambroise Paré (1510-1590) translated anatomical and battlefield experience into practical surgical technique. His writings and illustrations documented cranial instruments used for fracture management and trepanation, contributing to the standardization of instrument sets and operative steps [20]. Trepans, elevators, forceps, and lenticular instruments were increasingly adapted to specific tasks, reflecting the emergence of procedure-specific design rather than reliance on a single generic tool.

Technical refinement was driven by a recurring concern that remains recognizable to modern surgeons: how to remove enough bone to treat the pathology without causing additional injury. Historical analysis of trepan and trephine technique shows repeated emphasis on controlled drilling, probing the developing groove, avoiding plunge, and enlarging access with multiple openings when necessary [12]. In modern terms, this was an early form of risk engineering around the instrument-bone-dura interface.

The Renaissance did not yet solve the major biological hazards of surgery. Effective general anesthesia, germ theory, antisepsis, reliable hemostasis, and modern physiological support remained unavailable. Nevertheless, the period strengthened two prerequisites for safer cranial access: direct anatomical knowledge and specialized instrumentation. Those advances set the stage for the nineteenth-century convergence of localization, anesthesia, and infection control.

Enlightenment and the 19th century: from trephining to planned intracranial surgery

By the eighteenth century, trephining was an established component of surgical practice, but its indications were actively debated. John Bell’s 1795 Discourses on the Nature and Cure of Wounds discussed cranial trauma and the circumstances in which trepanation might or might not be justified [21]. Such debate reflected an important maturation of surgery: the technical ability to perform an operation no longer automatically implied that the operation should be performed.

Neuroanatomical and physiological advances reinforced this trend. Charles Bell’s work on the nervous system's anatomy and function became part of a broader 19th-century effort to relate neurological deficits to anatomical structures [22]. The emergence of cerebral localization later made it possible to plan a cranial opening according to the suspected location of pathology rather than relying only on external trauma.

Two developments fundamentally altered the conditions under which cranial surgery could be undertaken. In 1846, the public demonstration of ether anesthesia at Massachusetts General Hospital helped establish reliable surgical anesthesia, allowing longer and more controlled procedures without the movement and extreme pain that had constrained earlier operations [23]. In the second half of the 19th century, Joseph Lister’s antiseptic principles, followed by the development of aseptic surgery, reduced the burden of postoperative infection [24]. These innovations did not belong uniquely to neurosurgery, but neurosurgery could not have emerged as a viable specialty without them.

The clinical consequences became apparent in the late 19th century. William Macewen performed the first documented successful resection of a brain tumor in 1879, combining localization with antiseptic trephining [25]. Victor Horsley subsequently advanced surgery for epilepsy and intracranial tumors and became one of the central figures in the emergence of neurological surgery as a distinct field [26]. His historical interest in prehistoric trephining also illustrated how early neurosurgeons recognized continuity between ancient cranial openings and their own increasingly scientific practice [27].

This period therefore marked the transition from trephining as primarily an operation on the skull to craniotomy as a planned route to intracranial pathology. Anesthesia made time and stillness available; antisepsis made survival more likely; and localization made the opening purposeful. The remaining major obstacles were intracranial pressure, hemorrhage, and the technical handling of neural tissue (Figure 2).

**Figure 2:**
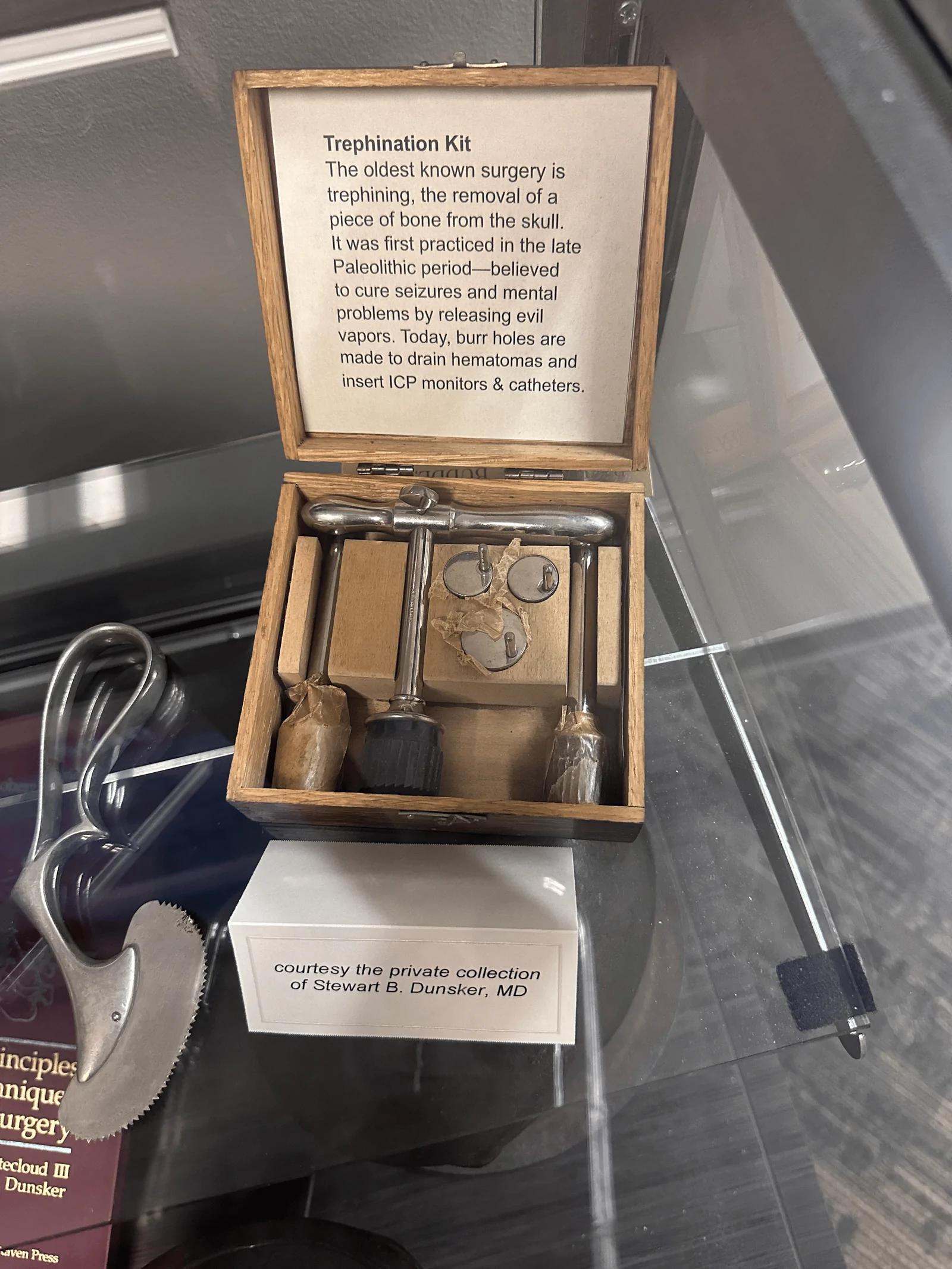
Historical trepanation instrument kit (18-19th century), including trephines of several sizes, brace, elevators, and accessory tools Image credit: Photograph by Natera-Rodriguez DE at Mayfield Clinic; reproduced with the author's permission.

Modern neurosurgery: from cranial access to controlled intracranial surgery

At the beginning of the twentieth century, the ability to open the skull was no longer the principal limiting step. The central problems were the physiological and technical consequences of operating within the intracranial compartment. Harvey Cushing’s early work illustrated this transition. At a time when the reported mortality for brain tumor surgery commonly ranged from 30% to 50%, Cushing’s integration of intracranial-pressure physiology, meticulous technique, and systematic clinical practice reduced mortality in his tumor series to less than 13% by 1910 [28]. His later work and that of his contemporaries addressed hemostasis, infection, and blood loss, including the development and adoption of technologies that made intracranial surgery more controlled [29].

Localization also became progressively technological. Walter Dandy introduced air ventriculography in 1918, using ventricular displacement to help localize intracranial lesions [30]. Although invasive by modern standards, ventriculography represented a conceptual breakthrough: the cranial opening could be planned in relation to an internal image rather than external landmarks alone. Subsequent cerebral angiography, computed tomography, and magnetic resonance imaging progressively transformed this relationship between anatomy, diagnosis, and surgical trajectory.

Modern craniotomy is now a routine access strategy for selected tumors, vascular lesions, hematomas, traumatic injuries, infections, epilepsy procedures, and other intracranial conditions [5]. Decompressive craniectomy represented a related but distinct principle in which bone is intentionally left off to create additional intracranial volume; contemporary reviews traced its lineage to ancient cranial decompression while emphasizing that modern indications and outcome assessment were fundamentally different [31].

Technological development has also reduced the amount of exposure required for many procedures. Operating microscopes, neuroendoscopy, stereotactic techniques, neuronavigation, intraoperative imaging, and image-guided keyhole approaches allow surgeons to tailor access to the target. Robotics, augmented and virtual reality, and artificial-intelligence-assisted planning are increasingly being investigated or integrated into neurosurgical workflows to improve visualization, trajectory planning, and instrument control [32].

The most recent historical literature places these advances within an even broader trajectory that includes military neurotrauma, evacuation systems, imaging, minimally invasive surgery, and modern multidisciplinary care [4]. The fundamental operation has therefore changed in meaning. A cranial opening is no longer primarily an end in itself; it is a precisely planned corridor whose size, location, and duration are dictated by the intracranial objective and by the effort to minimize collateral injury.

Discussion

The history of trepanation is most informative when it is not framed as a simple progression from superstition to science. Across different societies, cranial opening existed at the intersection of trauma, empirical observation, cultural belief, and available technology. A healed opening in a prehistoric skull and a modern image-guided craniotomy are separated by profound differences in knowledge and purpose, yet both address the same physical boundary: the skull as an obstacle to the treatment of intracranial disease or injury.

This distinction matters especially when assigning historical indications. Cranial trauma is strongly supported in some archaeological contexts, including Greek material associated with fractures [9,11]. Other cases lack an associated lesion that can identify a therapeutic target, and ritual or neurological explanations have been proposed [1,3]. These categories need not be mutually exclusive. A procedure can be both culturally meaningful and empirically therapeutic, and modern diagnostic categories cannot simply be projected backward onto skeletal material.

The same caution applies to “survival.” Archaeological studies typically infer survival from bone remodeling at the margin of the opening. This is a powerful observation because it shows that the patient lived after the procedure long enough for a biological response to occur. It does not establish the duration of survival with the precision of clinical follow-up, nor does it measure neurological status, infection, disability, or quality of life. For that reason, the 75%-83% estimates reported for Inca-period specimens and the historical American Civil War mortality estimates discussed by Kushner and colleagues should be viewed as historically illuminating but methodologically nonequivalent [3].

When this limitation is respected, a coherent pattern nevertheless emerges. Prehistoric practice demonstrated technical feasibility. Classical medicine added written classification and operative reasoning. Medieval scholarship preserved, translated, and modified technique. Renaissance anatomy and instrument design improved spatial control. Nineteenth-century anesthesia and antisepsis transformed the biological environment of surgery, while cerebral localization transformed the intellectual basis of targeting. Twentieth-century neurosurgery then confronted intracranial pressure, hemorrhage, tissue handling, and imaging. Contemporary technology increasingly focused on reducing the access burden itself.

This framework also clarified the relationship between trepanation, craniotomy, and craniectomy. They should not be treated as interchangeable terms. Trepanation described the broader historical act of creating a cranial opening; craniotomy described a planned temporary bone flap; and craniectomy described bone removal without immediate replacement [5]. The continuity among them was therefore conceptual rather than terminological: each represented a strategy for modifying the cranial boundary in service of an intracranial goal.

The educational value of this history lies in recognizing that modern neurosurgical safety is cumulative. Precise imaging is useful because anesthesia permits controlled surgery; anesthesia is useful because infection and hemorrhage can be managed; hemostasis is useful because anatomy and localization permit the surgeon to reach the correct target. No single innovation created modern craniotomy. Rather, successive layers of control converted an ancient act of access into a reproducible surgical platform.

Limitations

This vignette has several limitations inherent to its narrative and historical design. First, source selection was guided by relevance to major historical transitions rather than by a systematic-review protocol. This created the possibility of selection bias and omission of scholarship outside the databases, languages, and historical traditions examined. Historical literature also posed challenges of source verification and citation concordance because many claims persisted through secondary interpretations of primary texts, incomplete archival material, translation, and archaeological inference.

Second, much of the prehistoric evidence rested on skeletal findings. Osseous remodeling can support survival after cranial intervention but cannot independently establish the original indication, operative circumstances, neurological status, cultural meaning, or ultimate clinical outcome. The distinction between therapeutic, ritual, and post-mortem procedures was not certain in every specimen.

Third, quantitative estimates across eras were heterogeneous. Paleopathological survival inferred from bone healing is fundamentally different from clinically documented mortality. Small series, preservation bias, inconsistent definitions of healing, and uncertainty in chronological classification further limited direct comparison. Numerical data in this review were therefore presented to illustrate source-specific historical observations rather than to support pooled epidemiological conclusions.

Finally, organizing approximately 10 millennia of cranial surgical history into six broad periods necessarily compressed regional and cultural variation. The chronology should be interpreted as an explanatory framework for major changes in cranial access rather than as a uniform or strictly linear model of surgical progress.

## Conclusions

Trepanation is a durable marker of human attempts to treat disease and injury at the boundary between skull and brain. Its history demonstrates that the decisive evolution in cranial surgery was not simply the creation of an opening, but the accumulation of control around that opening. Prehistoric practitioners established that cranial access could be survived; classical authors formalized indications and technique; medieval scholars preserved and refined operative knowledge; Renaissance anatomy and instrumentation improved spatial understanding; nineteenth-century anesthesia, antisepsis, and localization made planned intracranial surgery feasible; and 20th- and 21st-century advances in physiology, hemostasis, imaging, microsurgery, navigation, endoscopy, robotics, and digital guidance have made access increasingly precise. Modern craniotomy is, therefore, not merely a technologically advanced descendant of trepanation. It is the product of thousands of years of progressively controlling why, where, how, and under what conditions the skull is opened. That continuity provides both historical perspective and a reminder of neurosurgery’s enduring objective: adequate access to intracranial pathology with the least possible biological cost.
